# Special Issue “Advances in Molecular Pathogenesis and Targeted Therapies for Myeloid Neoplasms”

**DOI:** 10.3390/ijms25042056

**Published:** 2024-02-08

**Authors:** Chung Hoow Kok, David T. Yeung, Devendra K. Hiwase

**Affiliations:** 1 Precision Cancer Medicine Theme, South Australian Health & Medical Research Institute (SAHMRI), Adelaide 5000, Australia; david.yeung@sa.gov.au; 2 Adelaide Medical School, University of Adelaide, Adelaide 5000, Australia; 3 Department of Genetics and Molecular Pathology, Centre for Cancer Biology, SA Pathology, Adelaide 5000, Australia; 4 Clinical and Health Sciences, University of South Australia, Adelaide 5000, Australia; 5 Department of Haematology, Royal Adelaide Hospital and SA Pathology, Adelaide 5000, Australia

Myeloid neoplasms (MNs) constitute a diverse group of haematological malignancies that includes myelodysplastic neoplasms (MDS), myeloproliferative neoplasms (MPN), MDS/MPN overlap syndrome, and acute myeloid leukaemia (AML). In this Issue, we collected articles that signified important advances in the field of multi-omics, providing novel insights into the disease biology, prognosis, and development of targeted therapies. Increasingly, however, these techniques also detect genomic variants in people not previously known to suffer from a haematological disorder. The next-generation sequencing (NGS) of >30,000 individuals without haematological abnormalities detected somatic mutations with variant allele frequency (VAF > 2%) in genes known to drive haematological malignancy. These were discovered in >10% of individuals older than 60 years of age [1,2,3]. These lesions, termed clonal haematopoiesis of indeterminate potential (CHIP), predominantly involves *DNMT3A*, *TET2*, and *ASXL1*, and is associated with increased risk of cardiovascular diseases, all-cause mortality, and haematological malignancy [1,2,3]. Although ageing-associated CH is known to drive a majority of myeloid neoplasms, 15–20% of myeloid neoplasms are considered to be direct and stochastic effects of DNA-damaging cytotoxic therapies used to treat antecedent conditions, most commonly primary cancers and/or autoimmune diseases. These therapy-related myeloid neoplasms (t-MN) are characterized by poor risk features, including complex karyotype, deletion 5q, deletion 7q, and *TP53* mutations [4]. Currently available therapies are largely ineffective in t-MN, which has a dismal five-year survival rate of <10%. Recent studies, including our own research, demonstrated the highly aberrant bone marrow microenvironment in myeloid neoplasms, particularly in therapy-related myeloid neoplasms, which may contribute to amplifying the oncogenic potential of the CH [5].

Certain patients may be more susceptible to the adverse late effects of DNA-damaging cytotoxic therapies. Genetic linkage studies of large numbers of patients provide novel insights into genetic predisposition to solid cancer and haematological malignancies. The screening of >10,000 adult cancer patients identified that 8% of patients harbour genetic predispositions to cancer; Ref. [6] in MN, this increases to 10–15% [7,8,9,10]. These mutations can shape clonal haematopoiesis, clinical features, and the progression to myeloid neoplasms [7,11,12,13]. The recognition of genetic predisposition aids in tracking clonal evolution, disease progression, treatment selection, and surveillance. For example, in a patient about to receive an allogeneic stem cell transplant, the identification of familial predisposition genes can optimise pre-transplant therapy, aid with donor selection (through avoiding the inadvertent use of related stem cell donor with the same germline mutation), provide information about the conditioning regimen and risk of graft versus host disease, and optimise post-stem cell transplant surveillance for additional cancers. For example, a patient with *DDX41* is more likely to respond to disease-modifying therapies such as lenalidomide and azacitidine, have higher risk of graft versus host disease, and display substantial risk of non-relapse mortality [14,15,16]. The identification of germline mutation in patients can also aid in genetic counselling for family members, open avenues for future surveillance, and provide intervention strategies.

Increasing awareness led to the inclusion of germline-mutated myeloid neoplasms as distinct entities in recent diagnostic classifications [17,18,19]. In this Special Edition, Paes et al. conducted a comprehensive review, focusing on the role of the *JAK2* (Janus Kinase 2) 46/1 haplotype in predisposition to MPN. The 46/1 haplotype (GGCC) comprises a collection of genetic variations distributed along chromosome 9p.24.1, spanning from the *JAK2* gene to insulin-like 4 (*INSL4*). It is characterized by the presence of four co-inherited variants (rs3780367, rs10974944, rs12343867, and rs1159782). This haplotype demonstrates a robust association with the development of *BCR::ABL1*-negative MPNs, and precedes the acquisition of the *JAK2* V617F, a common cause of MPNs. This haplotype appears to increase the risk of familial MPN by over fivefold, and is linked to various MPN-related clinical features, including inflammatory dysregulation, splenomegaly, splanchnic vein thrombosis, Budd–Chiari syndrome, as well as characteristic MPN-related changes. They propose that it may serve as a valuable biomarker for identifying individuals at high risk of developing MPN, potentially paving the way for novel strategies in the prevention and early detection of these disorders.

Acknowledging the prognostic importance of genetic changes, the fifth edition of World Health Organization (WHO) guidelines [17] and International Consensus Classification (ICC) [18] prioritize the classification of myeloid neoplasms, with greater emphasis on genetic changes over morphology. In this Special Issue, Zavras et al. reviewed research highlighting similarities and differences between high-risk MDS and AML with myelodysplasia-related changes (AML-MRC). AML-MRC is differentiated from de novo AML by the presence of specific chromosomal abnormalities (e.g., 5q, 7/7q, 20q deletions, and complex karyotypes) and somatic mutations (including *SF3B1*, *SRSF2*, *U2AF1*, *ZRSR2*, *STAG2*, *EZH2*, *BCOR*, *RUNX1*, *NRAS/KRAS*, *FLT3-ITD*, and *TP53*) which are also observed in MDS and have significant prognostic implications.

Furthermore, the development of a deeper understanding of the biology of high-risk MDS and the mechanisms of disease progression has paved the way for novel therapeutic approaches. These include combining venetoclax with hypomethylating agents and, more recently, the introduction of triplet therapies and agents targeting specific mutations, such as *FLT3* and *IDH1/2*. These advances are promising steps toward improving the management of patients with high-risk MDS and AML.

The therapeutic landscape of AML is rapidly changing after several decades of slow progress, with multiple novel therapies approved since 2017. A greater availability of targeted therapies leads to deeper molecular responses, longer disease-free duration, and overall survival. Measurable residual disease (MRD), determined by flow cytometry or molecular studies, is now more relevant than ever before in predicting prognosis of AML and guides decision making regarding haematopoietic cell transplantation. Consensus recommendations are now available from bodies such as the European LeukemiaNet [19,20,21]. Notably, recent regulatory approval for NPM1 MRD as a clinically relevant surrogate endpoint is poised to bring significant transformations to the manner in which clinical trials are conducted. This shift is expected to usher in biomarker-driven adaptive designs, offering a more refined and dynamic approach to AML therapy research and treatment evaluation. In this Special Issue, Tiong and Loo (2023) conducted a comprehensive review encompassing three key areas in AML MRD. Firstly, they explored emerging molecular markers for MRD assessment, including IDH1/2 and FLT3-ITD. Subsequently, they assessed the impact of novel therapeutic approaches on MRD endpoints in AML. Lastly, they delved into the potential utility of MRD as a predictive biomarker for guiding AML therapy, a focus of two significant collaborative trials: AMLM26 INTERCEPT (ACTRN12621000439842) and MyeloMATCH (NCT05564390).

Conventional treatments, such as chemotherapy, radiation, and stem cell transplantation, have been augmented by targeted therapies, immunotherapies, and hypomethylating agents, offering improved outcomes and a personalized approach to patient care. However, the emergence of treatment resistance poses a considerable challenge in managing MN. Resistance to therapy, often driven by genetic mutations and molecular alterations, can lead to treatment failure, disease relapse, and poor prognosis. Understanding and anticipating treatment resistance is of paramount importance to further improving outcomes.

The realisation that BCL-2 is overexpressed in myeloid neoplasms has led to the use of venetoclax, a potent and selective BCL-2 inhibitor in AML. Venetoclax binds to BCL-2, blocking its anti-apoptotic effects and promoting programmed cell death. However, the duration of response to venetoclax and combinations thereof is often limited, often resulting in leukaemia relapse and treatment failure. In a study conducted by Seipel et al., they assessed the effectiveness of various pro-apoptotic agents in vitro, including the BCL-XL inhibitor A1331852, the MCL1 inhibitor S63845, the dual PI3K-mTOR inhibitor bimiralisib (PQR309), the BMI-1 inhibitor unesbulin (PTC596), the MEK inhibitor trametinib (GSK1120212), and the STAT3 inhibitor C-188-9, both as single agents and in combination with venetoclax. They evaluated these combinations for their ability to induce apoptosis and cell death in leukemic cells, with or without bone marrow stroma support. Their findings showed that all combination treatments with venetoclax exhibited enhanced cytotoxic effects in AML cell lines and AML patient samples. Notably, cells from patients with *IDH2* and *FLT3* mutations were particularly sensitive to the venetoclax and bimiralisib combination, whereas non-responders were associated with *PTPN11* mutations. Such a combination of dual PI3K/mTOR pathways and BCL-2 inhibition is a promising approach.

Spleen tyrosine kinase (SYK) is a crucial protein involved in the survival and proliferation of AML cells. SYK is highly expressed by haematopoietic cells and has emerged as a potential therapeutic target. Brattas et al. investigated the in vitro efficacy of five SYK inhibitors—fostamatinib, entospletinib, cerdulatinib, TAK-659, and RO9021—using leukemic blasts from a consecutive cohort of AML patients. These inhibitors exerted a concentration-dependent antiproliferative effect on AML cells. Particularly, fostamatinib and TAK-659 demonstrated significantly higher antiproliferative effects in patients with FLT3 mutations compared to the wild type. The SYK inhibitors led to a significant reduction in the release of cytokines and chemokines from primary AML cells, signifying their potent inhibitory effect on these crucial leukemic signalling molecules. Further studies are needed to determine which specific subsets of AML patients are likely to benefit from SYK inhibitors.

Azacitidine (AZA) is a frequently utilized hypomethylating agent in the treatment of higher-risk MDS and AML. While some patients do achieve remission with AZA therapy, a significant portion eventually experiences treatment failure. To establish a comprehensive understanding of the mechanisms underlying AZA resistance, Kutnya et al. conducted a thorough analysis encompassing intracellular uptake and retention (IUR) of radiolabeled AZA (^14^C-AZA), gene expression, transporter pump activity with or without inhibitors, and cytotoxicity in both treatment-naïve and resistant cell lines. Notably, the cytoplasmic concentration of ^14^C-AZA was markedly reduced in resistant cells (MOLM-13 and SKM-1) compared to their respective parental cells, correlating with a progressive decrease in the expression of *SLC29A1*, a cellular influx transporter. Conversely, the study did not find changes in the expression of cellular efflux pumps like ABCB1 and ABCG2 in AZA-resistant cells, suggesting that these pumps are unlikely to contribute to AZA resistance. This study establishes a causal connection between in vitro AZA resistance and the downregulation of the cellular influx transporter *SLC29A1*.

Taken together, these recent advances are bringing us closer to a future where tailored treatments based on the unique genetic and molecular profiles of individual patients may become the new standard of care for MN, ultimately offering the promise of improved outcomes and a better quality of life for those affected by MN.

## Data Availability

Not applicable.
